# Vitamin D Uptake in Patients Treated with a High-Dosed Purified Omega-3 Compound in a Randomized Clinical Trial Following an Acute Myocardial Infarction

**DOI:** 10.3389/fcvm.2017.00041

**Published:** 2017-07-24

**Authors:** Patrycja A. Naesgaard, Heidi Grundt, Arne F. Nordøy, Harry Staines, Dennis W. T. Nilsen

**Affiliations:** ^1^Department of Cardiology, Stavanger University Hospital, Stavanger, Norway; ^2^Department of Clinical Science, University of Bergen, Bergen, Norway; ^3^Department of Medicine, Stavanger University Hospital, Stavanger, Norway; ^4^Department of Medicine, Institute of Clinical Medicine, University of Tromsø, Tromsø, Norway; ^5^Sigma Statistical Services, Balmullo, United Kingdom

**Keywords:** vitamin D, omega-3, acute myocardial infarction, 25-hydroxyvitamin D, omega-6

## Abstract

**Background:**

Fish is the natural dietary source of vitamin D. Reports on the influence of purified omega-3 fatty acids on its uptake are scarce.

**Objectives:**

We investigated the impact of a purified high-dose omega-3 compound compared to corn oil on 25-hydroxyvitamin D [25(OH)D] levels following an acute myocardial infarction.

**Methods:**

228 patients were randomized 1:1 to receive a daily dose of either 4 g omega-3 (OMACOR^®^) or an equal dose of corn oil, administered double-blindly for 12 months. Total omega-3 and omega-6 measurements were available in 40 randomly picked patients.

**Results:**

There was no significant intergroup difference in 25(OH)D changes at 12 months follow-up (*p* = 0.12), but there was a minor statistical significant intragroup increase in 25(OH)D in both intervention arms (*p* < 0.001 for n-3 polyunsaturated fatty acids and *p* = 0.013 for corn oil, respectively). A positive correlation was noted between 25(OH)D and omega-3 prior to inclusion; *r* = 0.418, *p* = 0.007, attenuated at 12 months by purified omega-3 intervention; *r* = 0.021, *p* = 0.93. No positive correlation was observed between omega-6 and 25(OH)D.

**Conclusion:**

Long-term treatment with a high dose of purified omega-3 as compared to corn oil did not improve serum concentrations of vitamin D.

**Clinical Trial Registration:**

ClinicalTrials.gov, Identifier: NCT01422317.

## Introduction

Vitamin D can be synthesized in the human epidermis on exposure to ultraviolet light or it can be ingested mainly through consumption of oily fish (1). Thus, low levels of vitamin D can be caused by limited sun exposure and/or inadequate intake from the diet. Individual differences in vitamin D baseline concentration, absorption, and metabolism may also influence its level in humans.

Previous studies indicate that different vehicles including n-3 polyunsaturated fatty acids (PUFAs) may influence the bioavailability of vitamin D (2). These studies have evaluated different patient populations, different vitamin D supplementations, and duration of intake.

We have previously shown that serum levels of 25-hydroxyvitamin D [25(OH)D] may predict total and cardiac mortality in chest pain patients with suspected acute coronary syndrome (3). Several other studies have demonstrated an inverse relationship between cardiovascular risk and levels of 25(OH)D and omega-3 (4, 5). As 25(OH)D concentrations are generally found in the lower range of recommended levels (3), it is important to define all steps that may influence vitamin D intake, absorption, and deposition, in order to enforce recommendations as a preventive measure in relation to disease.

To determine whether a purified compound of omega-3 may positively influence the uptake of vitamin D, this would scientifically require a randomized study with purified omega-3 supplementation as compared to a regular diet containing an equivalent amount of naturally occurring omega-3. Furthermore, such a study may not be acknowledged due to its open design. Another possibility would be to examine the influence of long-term treatment with a purified omega-3 compound on the association between omega-3 and vitamin D.

In 1990s, we performed a randomized, double-blind study including 300 patients with an acute myocardial infarction (MI), treated with a high dose of purified omega-3 as compared to corn oil (6). Serum levels of total omega-3 and omega-6 prior to and at completion of intervention were determined in a subset of patients (7).

It has previously been demonstrated that the uptake of a vitamin D supplement dissolved in fish oil may not differ as compared to vitamin D administered as a multivitamin tablet, during an intervention period of 4 weeks (8). However, information related to long-term supplementation with omega-3 as compared to corn oil free of 25(OH)D with respect to vitamin D uptake from the diet has previously not been elucidated.

The primary aim of the present study was to evaluate whether a purified omega-3 compound (ethylester form) as compared to corn oil would increase the uptake of vitamin D provided in the diet.

Thus, in the present study we have retrospectively measured serum vitamin D levels as 25(OH)D in 228 patients hospitalized with a MI with available blood samples at baseline and during follow-up.

The second aim was to assess the correlation between 25(OH)D and omega-3 and omega-6 fatty acids (FAs), respectively. This was performed in 19 and 21 randomly picked patients from the respective groups.

The present study was based on the hypothesis that a purified compound of omega-3 not containing vitamin D would have a limited impact on vitamin D concentrations.

## Materials and Methods

**Figure 1:**
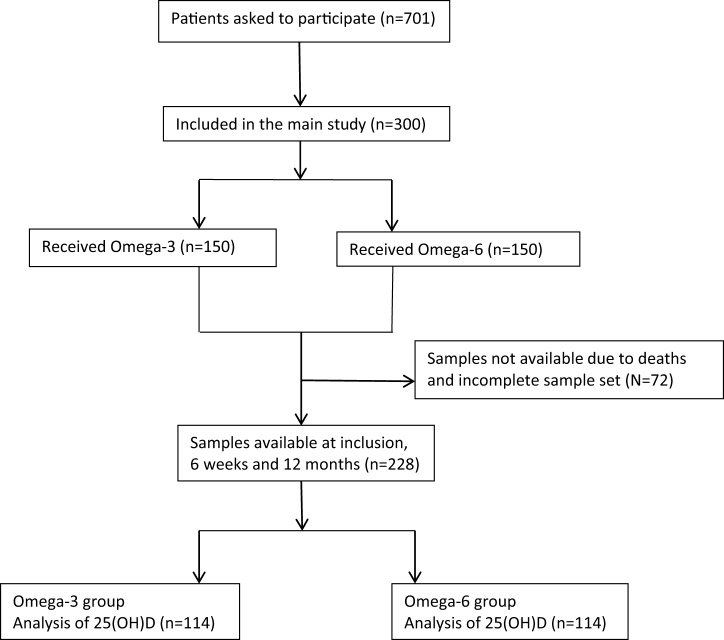
Flow chart.

Participants in the present analysis belonged to the Omacor Following Acute Myocardial Infarction (OFAMI) study (ClinicalTrials.gov Identifier: NCT01422317), who were hospitalized with a MI at Central Hospital in Rogaland, Stavanger, Norway from September 1995 until December 1996 and randomly assigned 1:1 in blocks of four to a daily dose of either 4 g highly concentrated omega-3 FA, containing 85% eicosapentaenoic acid and docosahexaenoic acid (OMACOR™, Pronova A/S, Oslo, Norway), or to corn oil (basically n-6 FAs), administered double blindly for at least 12 months. 4 mg of alpha-tocopherol was added to each capsule to protect against FA oxidation. Eligibility was based on several exclusion criteria, including liver dysfunction (6). A flow chart providing information related to recruitment and inclusion in the present study is presented in Figure 1. No significant amendments were made after trial commencement.

The diet contained a high intake of fish meals; two meals or less in the lower quartile and three meals or more in the upper quartile. The median intake consisted of three fish meals per week. Dietary habits remained essentially unchanged throughout the study period, whereas supplementary fish oil products were discontinued immediately following inclusion in the trial (6, 7).

Blood samples for the present analysis were available in 228 OFAMI patients. 25(OH)D was measured at baseline, 6 weeks, and 12 months follow-up in these patients (114 patients received omega-3 FAs and 114 patients received corn oil, and number of fish meals was similar in both groups). In a substudy previously reported by Grundt et al. (9), total omega-3 and omega-6 FAs, respectively, were measured in serum phospholipids in samples from 60 randomly picked patients. These analyses were performed during the study period at baseline and after 12 months of treatment. In this subpopulation, we investigated the association between FA and 25(OH)D levels.

Blood samples were drawn following admission, and serum for measurement of 25(OH)D was stored at −80°C for later analysis. 25(OH)D analysis was performed at the Department of Medical Biochemistry, Stavanger University Hospital, by determination of the metabolites 25(OH)D_3_ and 25(OH)D_2_ in serum by liquid–liquid extraction, derivatization with 4-phenyl-1,2,4-triazoline-3,5-dione reagent (Sigma-Aldrich, St. Louis, MO, USA), and analysis by liquid chromatography coupled with tandem mass spectrometry detection, as previously described (3). The long-term stability of 25(OH)D serum concentrations is maintained under these conditions (10).

Analyses of FAs in serum phospholipids were carried out at the University of Tromsø, Norway, as previously reported (11).

The study was approved by the Regional Board of Research Ethics and the Norwegian Health Authorities and conducted in accordance with the Declaration of Helsinki of 1971, as revised in 1983. Written informed consent was obtained from all patients. The details of the OFAMI study have been published previously (6).

### Statistical Analysis

Changes in 25(OH)D from baseline to 6 weeks and 12 months follow-up, respectively, were calculated for each individual patient. The paired *t*-test was used to evaluate whether changes in parameter values from baseline to follow-up were significantly different from zero. The independent two-sample *t*-test was used to test for differences between the two treatment groups. Approximately normally distributed variables were given as mean and standard deviation (SD).

Spearman’s correlation coefficients were calculated between 25(OH)D and total omega-3 and omega-6 FAs, respectively, and a two-tailed test from zero was applied.

The statistical analyses were performed using the statistical package SPSS version 19. All tests were two-sided with a significance level of 5%. Highly significant differences were based on a two-tailed significance level of 1%.

The present study was based on the hypothesis that a purified compound of omega-3, not containing vitamin D, would have a limited impact on vitamin D concentrations. If a minor influence should be present, an increase below 5 nmol/L as compared to controls would be considered of less clinical relevance.

Given an SD of 12, and a 5% significance level, then 114 patients in each group gives 99.3% power to detect a mean of 5 nmol/L in the change from baseline within each group and 87.9% power to detect a mean difference of 5 nmol/L between the treatment groups.

## Results

### Baseline Data

A total of 228 available subjects from the OFAMI study (6) were included in the present analysis and followed up at 6 weeks and 12 months. In the present population, there were 186 men (61.6 ± 12.4 years) and 42 women (67.8 ± 11.0 years), equally divided between the two treatment groups. Patient characteristics at baseline according to treatment groups are presented in Table 1. There were no significant intergroup differences at baseline.

**Table 1:** Patient characteristics at baseline according to treatment group.

Characteristics (*n*)	n-3 group (*n* = 114)	Corn oil group (*n* = 114)	*p*-Value
Male sex	93	93	0.86
Current smoker	46	43	0.78
STEMI	47	51	0.69
Thrombolysis	46	50	0.69
Diabetes mellitus	14	10	0.52
Heart failure	8	5	0.57
Hypertension	25	26	0.88
EF < 30%	8	5	0.57
EF 30–50%	35	39	0.67

*STEMI, ST elevation myocardial infarction; EF, ejection fraction*.

### Interventional Study

Mean (±SD) 25(OH)D levels in the two groups at baseline, 6 weeks, and 12 months on treatment are shown in Table 2. The increase in 25(OH)D was statistically significant in both treatment groups at 12 months (*p* < 0.001 for n-3 PUFAs and *p* = 0.013 for corn oil, respectively). There was no significant intergroup difference between changes in 25(OH)D concentrations after 6 weeks (*p* = 0.61) and 12 months intervention (*p* = 0.12).

**Table 2:** 25(OH)D nmol/L levels at baseline, after 6 weeks, and after 12 months supplementation with high dose of n-3 PUFAs (*n* = 114) as compared to corn oil (*n* = 114).

25(OH)D nmol/L mean (±SD)			
	Baseline	6 weeks	12 months
n-3 PUFAs (*n* = 114)	51.25 ± 15.15	52.66 ± 14.57	56.22 ± 17.77**
Corn oil (*n* = 114)	54.82 ± 17.78	55.64 ± 17.82	57.43 ± 19.16*

*No significant difference between groups at baseline, *p* = 0.10 (independent *t*-test)*.

**^p^ < 0.001 as compared to baseline (paired t-test).

*^p^ = 0.013 as compared to baseline (paired t-test).

*No significant difference between intergroup changes after 6 weeks intervention (p = 0.61) or after 12 months intervention (p = 0.12)*.

*25(OH)D, 25-hydroxyvitamin D; PUFA, polyunsaturated fatty acid*.

The FA profiles did not differ between the treatment groups at baseline. In the group receiving n-3 PUFAs, the total amount of n-3 FAs increased significantly as compared to the group receiving n-6 PUFAs (*p* = 0.02), as shown in Table 3.

**Table 3:** Changes in biomarkers related to a subpopulation with known serum concentrations of fatty acids and 25(OH)D [mean (SD), *n* = 40].

	n-3 group (*n* = 19)		Corn oil group (*n* = 21)	
	Baseline	12 months	Baseline	12 months
Total n-3 PUFAs (μmol/L)^2^	285.41 (85.390)	466.016 (96.986)**	326.352 (131.588)	367.933 (155.642)
Total n-6 PUFAs (μmol/L)^1^	1,185.690 (312.469)	1,052.963 (177.628)	1,283.400 (281.854)	1,348.552 (231.660)
Total saturated FAs (μmol/L)	2,020.584 (440.313)	2,000.258 (229.395)	2,153.091 (393.898)	2,205.952 (305.543)
Ratio n-3/n-6^2^	0.2514 (0.0887)	0.458 (0.132)**	0.2631 (0.1179)	0.282 (0.1323)
Cholesterol (mmol/L)	5.47 (0.98)	5.24 (0.79)	5.93 (1.13)	5.76 (0.91)
HDL-cholesterol (mmol/L)	0.96 (0.18)	1.22 (0.26)**	1.17 (0.35)	1.24 (0.35)
Triglycerides (mmol/L)	1.78 (1.09)	1.35 (0.51)	1.43 (0.63)	1.73 (0.78)
Vitamin D [25(OH)D] (nmol/L)	54.275 (16.104)	57.409 (20.392)	58.575 (21.734)	59.106 (22.605)

Intragroup difference from baseline to 12 months; **p < 0.001.

*No difference between groups at baseline, except for HDL cholesterol, p = 0.029*.

*Significance of difference in change from baseline between groups: ^1^p = 0.05, ^2^p < 0.001*.

*25(OH)D, 25-hydroxyvitamin D; PUFA, polyunsaturated fatty acid*.

### Correlation Study

The correlation between 25(OH)D and omega-3 FAs prior to inclusion was found to be statistically significant (*r* = 0.418, *p* = 0.007), whereas no statistically significant correlation was found between 25(OH)D and omega-6 FAs (*r* = −0.073, *p* = 0.66). At 12 months intervention with n-3 PUFAs the correlation between 25(OH)D and omega-3 and omega-6, respectively, was no longer significant (*r* = 0.021, *p* = 0.93 and *r* = 0.163, *p* = 0.51, respectively). At 12 months intervention with corn oil, the omega-6 was negatively correlated with 25(OH)D (*r* = −0.469, *p* = 0.032). These correlation results are presented in Table 4.

**Table 4:** Spearman correlation coefficients between 25(OH)D and fatty acids at baseline and after 12 months intervention with PUFAs or CO.

FAs	Baseline (*n* = 40)		12 months intervention with PUFAs (*n* = 19)		12 months intervention with CO (*n* = 21)	
			
	*r*	*p*-Value	*r*	*p*-Value	*r*	*p*-Value
Total saturated FAs	0.057	0.73	0.205	0.40	−0.077	0.74
Total n-3 PUFAs	0.418	0.01	0.021	0.93	0.482	0.03
Total n-6 PUFAs	−0.073	0.66	0.163	0.51	−0.469	0.03

*25(OH)D, 25-hydroxyvitamin D; FAs, fatty acids; CO, corn oil; PUFAs, polyunsaturated fatty acids*.

## Discussion

In the present population consisting of a total of 228 subjects with an acute MI, there was a minor, but statistically significant increase in levels of 25(OH)D at 12 months in both treatment groups. However, there was no significant difference in vitamin D concentrations in the omega-3 group as compared to the corn oil group of patients (*p* = 0.12). Whether the improvement in both groups is a consequence of intervention or whether it reflects post-MI dietary changes cannot be determined. Although purified compounds of omega-3 do not contain vitamin D, treatment with such compounds might enhance vitamin D absorption from other food sources and/or might upregulate vitamin D receptors. In the present patient material, vitamin D levels were found to be suboptimal as compared to recommended concentrations above 75 nmol/L, as shown in Table 2. As the values are fairly low, we might expect an increased influence of omega-3 on the uptake of vitamin D from the diet. We found a statistically non-significant intergroup difference in vitamin D levels of 2.4 nmol/L. Even if this difference had been statistically different, its clinical relevance would be negligible according to our hypothesis. Furthermore, although the omega-3 levels in our patients prior to intervention were high as compared to other populations (6), vitamin D levels were low, lending support to our assumption.

The background diet of the present population was rich in omega-3, allowing us to investigate the relation between dietary omega-3 and vitamin D at baseline. Prior to intervention we observed a statistically significant positive correlation between omega-3 and 25(OH)D. However, after 12 months intervention with a high dose of purified omega-3, the positive correlation between omega-3 and 25(OH)D was lost. Dietary omega-6 was not found to correlate with 25(OH)D and intervention with corn oil resulted in a negative correlation.

These results may support previous findings related to a healthy population, in which 25(OH)D was positively associated with total daily monounsaturated FAs intake and inversely associated with total PUFAs intake, usually consisting of a high proportion of n-6 as compared to n-3 FAs (12). Moreover, Itariu et al. (13) employed a comparable dose of OMACOR™ to that of the present study for 8 weeks and found that purified n-3 PUFAs did not affect vitamin D status, which is largely in agreement with our findings.

Overall, these results would suggest that the increase in vitamin D in our two intervention groups may rather be related to a recovery situation, including dietary and lifestyle changes following an acute MI and not to the intervention *per se*. Furthermore, our correlation findings indicate that omega-3 may not act alone as a vehicle for vitamin D absorption, as demonstrated by the attenuation of the correlation between omega-3 and vitamin D following intervention with a purified compound of omega-3.

Holvik et al. (8) have shown that the uptake of vitamin D did not depend on whether vitamin D was given as a multivitamin tablet or as a fish oil capsule, both containing the same amount of vitamin D. Essentially, similar results were obtained by Tangpricha et al. (14), who studied three different vehicles with vitamin D fortification. This would also support our findings that vitamin D is independently associated with omega-3 FAs in diets containing fish.

No negative correlation was observed in the omega-3 intervention group, whereas the positive correlation at baseline was inversed by 12 months of corn oil intervention. This would imply that the latter compound may negatively influence the relationship between vitamin D and omega-3. These results are in agreement with those of Olsen et al. (15), who investigated the associations of plasma vitamin D, marine PUFAs, and PUFA ratios in women from the Norwegian Women and Cancer Post-Genome Cohort. In that study, vitamin D levels were found to be weakly associated with PUFA ratios, mainly containing omega-6 FAs, but significantly associated with marine FAs.

Although vitamin D is positively correlated with omega-3 in the background diet, attenuation of the vitamin D levels by purified n-3 PUFAs may indirectly suggest the coexistence of additional vehicles for vitamin D absorption in a marine diet. This assumption is supported by results of dietary intervention studies such as DART 1 (16), that of Kromhout et al. (17), and JPHC (18), in which a diet rich in natural fish oils was found to be beneficial in relation to clinical outcome, whereas other studies, such as ORIGIN (19), using purified omega-3 oils have shown neutral results on outcome.

The GISSI Prevention trial (20) and the GISSI HF study (21) have demonstrated clinical benefits of OMACOR™ 1 g per day, whereas ORIGIN (19) did not, and we are still awaiting the results of the ASCEND study (22). Also, other large intervention studies, such as JELIS (23), claim a benefit of purified omega-3, but results of meta-analyses (24, 25) still remain controversial with respect to the clinical benefit of purified omega-3 compounds. In dietary studies, claiming a beneficial effect of omega-3, vitamin D may contribute to the improved prognosis.

The present study was designed to evaluate the association between vitamin D and different PUFAs, administered as either omega-3 or corn oil, and not to investigate the clinical performance of these PUFAs. Our findings suggest that vitamin D may be an independent player of importance in an omega-3 rich diet.

### Limitations

Our study was performed retrospectively. The analyses of vitamin D and FAs in serum phospholipids were performed without knowledge of treatment regimen.

The blood samples were stored for more than 10 years before the measurement of vitamin D. However, it has been shown that vitamin D is stable and will not be significantly affected by long-term storage (10).

Our correlation study suggests an attenuating effect of both purified PUFA compounds on vitamin D, which is indirect evidence and should be considered with caution.

In conclusion, high doses of purified omega-3 FAs have a low impact on the levels of vitamin D and weaken the correlation between omega-3 and vitamin D.

## Ethics Statement

This study was carried out in accordance with the recommendations of Directive 95/46/EC of the European Parliament and of the Council of October 24, 1995 on the protection of individuals with regard to the processing of personal data and on the free movement of such data, Regional Committee for Medical and Health Research Ethics, Western Norway with written informed consent from all subjects. All subjects gave written informed consent in accordance with the Declaration of Helsinki. The protocol was approved by the Regional Committee for Medical and Health Research Ethics, Western Norway.

## Author Contributions

PN: contributed to study design, data collection, vitamin D analysis, interpretation of results, and preparation of the manuscript. HG: contributed to data collection, clinical follow up, interpretation of results, and preparation of the manuscript. AN: contributed to study design, interpretation of the results, and commented on the manuscript. HS: performed the statistical analysis, contributed to the interpretation of the results, and commented on the manuscript. DN: conceived the idea of the study, supervised the study including interpretation of results, and preparation of the manuscript. All the authors have read and approved the final manuscript.

## Conflict of Interest Statement

The authors declare that the research was conducted in the absence of any commercial or financial relationships that could be construed as a potential conflict of interest.
